# Taxifolin and Sorghum Ethanol Extract Protect against Hepatic Insulin Resistance via the miR-195/IRS1/PI3K/AKT and AMPK Signalling Pathways

**DOI:** 10.3390/antiox10091331

**Published:** 2021-08-24

**Authors:** Hana Lee, Won-Tae Jeong, Yoon-Sup So, Heung-Bin Lim, Junsoo Lee

**Affiliations:** 1 Department of Food Science and Biotechnology, Chungbuk National University, Cheongju 28644, Korea; dlgksk0514@naver.com; 2 Residual Agrichemical Assessment Division, National Institute of Agricultural Science, Rural Development Administration, Wanju 55365, Korea; shewaspretty@korea.kr; 3 Department of Crop Science, Chungbuk National University, Cheongju 28644, Korea; yoonsupso@chungbuk.ac.kr; 4 Department of Industrial Plant Science & Technology, Chungbuk National University, Cheongju 28644, Korea; heungbin@chungbuk.ac.kr

**Keywords:** microRNA, insulin resistance, sorghum, taxifolin, miR-195

## Abstract

This study aimed to evaluate the effects of taxifolin and sorghum ethanol extract on free fatty acid (FFA)-induced hepatic insulin resistance. FFA treatment decreased glucose uptake by 16.2% compared with that in the control, whereas taxifolin and sorghum ethanol extract increased the glucose uptake. Additionally, taxifolin and sorghum ethanol extract increased the expression of p-PI3K, p-IRS1, p-AKT, p-AMPK, and p-ACC in FFA-induced hepatocytes. Furthermore, FFA treatment increased the expression of miR-195. However, compared with the FFA treatment, treatment with taxifolin and sorghum ethanol extract decreased miR-195 expression in a dose-dependent manner. Taxifolin and sorghum ethanol extract enhanced p-IRS1, p-PI3K, p-AMPK, p-AKT, and p-ACC expression by suppressing miR-195 levels in miR-195 mimic- or inhibitor-transfected cells. These results indicate that taxifolin and sorghum ethanol extract attenuate insulin resistance by regulating miR-195 expression, which suggests that taxifolin and sorghum ethanol extract may be useful antidiabetic agents.

## 1. Introduction

Sorghum is an important cereal grain in Asian countries for human consumption and potential health-promoting properties [1,2]. Phenolics isolated from sorghum promote beneficial changes in diseases such as diabetes, obesity, hypertension, and cancer [3,4,5]. Taxifolin (dihydroquercetin) is a bioactive flavonoid found in sorghum grains. Taxifolin has antihypertensive, antioxidant, and antitumor activities [6,7,8]. Taxifolin alters the expression levels of several genes such as those encoding detoxification enzymes, growth factors, and cell cycle regulatory proteins [9]. Teselkin et al. (2000) showed that taxifolin offered anti-oxidative defenses in rats with tetrachloromethane-induced hepatitis [10].

The liver is an important organ for glucose production and utilization as well as metabolic homeostasis. Insulin resistance leads to hyperglycaemia and abnormalities in the hepatic glucose output [11]. Free fatty acids (FFAs) have been shown to directly impair insulin signaling in hepatocytes [12]. In general, two main signaling pathways are involved in the regulation of glucose metabolism: the insulin receptor substrate-1 (IRS1)/phosphoinositide 3-kinase (PI3K)/protein kinase B (AKT) and AMP-activated protein kinase (AMPK) pathways [13]. IRS1 has been linked to the treatment of hepatic insulin resistance [14]. Phosphorylation of IRS1 activates PI3K, regulates the downstream factor AKT, and promotes glucose uptake [15]. Activation of AMPK coordinates glucose metabolism by regulating gluconeogenesis and glycolysis [16]. Additionally, AMPK can enhance ATP-generating catabolic pathways, including lipolysis, unsaturated fat oxidation, and glucose uptake [17]. Therefore, a comprehensive understanding of the underlying insulin resistance mechanism and the development of treatment strategies are important for the prognosis of diabetes.

MicroRNAs (miRs) are short and non-protein-coding gene products that can modulate gene expression through post-transcriptional inhibition of mRNA translation by binding to the 3′-untranslated regions (3′-UTRs) of target gene mRNA [18]. The interaction of the miR seed sequence and 3′-UTRs of specific mRNAs regulate multiple diverse biological processes such as insulin secretion and neurodevelopment [19]. miR-375 overexpression resulted in decreased insulin sensitivity and β-cell number, both of which were reversed by miR-375 inhibition [20]. In addition, miR-320 can regulate insulin resistance in 3T3-L1 adipocytes by targeting PI3K [21]. Shen et al. (2017) reported that FFA treatment increased miR-34-3p, miR-21-3p, miR-21-5p, and miR-451a, however, liraglutide decreased these miRs [22]. A previous study showed that insulin resistance induced by a saturated fatty acid upregulates miR-195 in HepG2 cells [23]. In addition, in a spontaneous rat model of type 2 diabetes (T2D), miR-195 expression levels correlating with glycaemic status were upregulated [24]. Karolina et al. (2012) identified miR-195 as potential contributors in patients with metabolic syndrome and T2D [25]. These findings indicate that miR-195 plays an important role in insulin resistance. However, the antidiabetic activity of taxifolin and sorghum ethanol extract through miR-195 regulation and its association with the IRS1/PI3K/AKT and AMPK pathways remain unclear. Therefore, we aimed to evaluate the mechanisms underlying hepatic insulin resistance via miR-195 modulation.

## 2. Materials and Methods

### 2.1. Materials

Sorghum (*Sorghum bicolor* (L.) Moench) was provided by the Rural Development Administration (Suwon, Gyeonggido, Korea). Taxifolin, fatty acid-free bovine serum albumin (BSA), sodium palmitate, and sodium oleate were obtained from Sigma-Aldrich (St. Louis, MO, USA). Antibodies against p-IRS1, IRS1, p-PI3K, PI3K, p-AKT, AKT, p-AMPK, AMPK, p-ACC, ACC, and β-actin were obtained from Cell Signaling Technology (Danvers, MA, USA) and Santa Cruz Biotechnology (Santa Cruz, CA, USA).

### 2.2. Ultra-Performance Liquid Chromatography-Electrospray Ionization Quadrupole Time-of-Flight Mass Spectrometry (UPLC-ESI-Q-TOF/MS)

The UPLC system (ACQUITY UPLC System, Waters Corp., Worcester, MA, USA) consisted of a binary solvent delivery pump, auto-sampler, and photodiode array (PDA). Q-TOF/MS was performed according to a previously described method using Xevo G2 Q-TOF/MS (Waters Corp., Worcester, MA, USA) coupled with an ESI source. [26]. UNIFI (verion 1.8) software (Waters Corp.) was used to acquire and process the data. The analytes were detected in negative ion mode, and the capillary and cone voltages were set to 3.0 kV and 30 V, respectively. The cone and desolvation gas flow rates were set to 60 and 800 L. The cone and desolvation gas flow rates were set to 60 and 800 L h^−1^, respectively. The temperature of the source was 120 °C. The scan time was set to 0.25 s in the range of 50–1200 *m*/*z*. To acquire the mass spectrum and MS/MS, the low and high collision energies were set to 6 and 30–50 eV, respectively.

### 2.3. Free Fatty Acid Mixture Preparation

FFAs were mixed with 10% fatty acid-BSA, as described previously [27], and 4 mM sodium palmitate and 8 mM sodium oleate stock solution were prepared in 50 mM NaOH by heating at 70 °C. Additionally, 10% BSA solution was prepared in ddH_2_O and maintained at 55 °C in a water bath, and 10 mM FFA/1% BSA solution was obtained by complexing the appropriate amount of palmitate and oleate stock solution to 10% BSA at 55 °C for another 30 min. The solution was then cooled to 25 °C, filter sterilized, and stored at −20 °C until use.

### 2.4. Cell Culture and Cytotoxicity

Human-derived hepatic cell lines (HepG2; HB-8065, ATCC, Manassas, VA, USA) were maintained in Dulbecco’s modified Eagle’s medium with 10% heat-inactivated foetal bovine serum and 1% penicillin-streptomycin at 37 °C in 5% CO_2_ humidified air. Cytotoxicity was evaluated by the MTT assay. HepG2 cells were seeded onto plates at a density of 1 × 10^5^ cells/mL and treated with an FFA mixture (500 μM) for 18 h. After FFA exposure, the hepatocytes were immediately treated with taxifolin or sorghum ethanol extract in RPMI1640 containing 0.2% BSA for an additional 24 h. After culture, MTT reagent (1 mg/mL) was added to each well and incubated for 2 h. The medium was removed, and the blue crystalized formazans were dissolved in DMSO. Absorbance at 550 nm was measured using a microplate reader (BioTek, Inc., Winooski, VT, USA).

### 2.5. Glucose Uptake

Glucose uptake assay was performed as stated before with slight modifications [28]. HepG2 cells were cultured in a serum-free medium containing an FFA mixture with taxifolin or sorghum ethanol extract for 18 h and then treated with taxifolin or sorghum ethanol extract in RPMI 1640 containing 0.2% BSA and insulin (100 nM) for 15 min. The culture medium was harvested and glucose colorimetric assay kit II (BioVision, Inc., San Francisco, CA, USA) was used. Absorbance at 450 nm was measured (BioTek, Inc., Winooski, VT, USA).

### 2.6. Western Blotting

Protein expression levels were confirmed as previously described [29]. Equal amounts of proteins were separated on a 10% sodium dodecyl sulphate polyacrylamide gel and electrophoretically transferred to a nitrocellulose membrane (GE Healthcare, Buckinghamshire, UK). The membranes were blocked with Tris-buffered saline/Tween 20 (TBST) containing 5% skim milk and incubated for 12 h with primary antibodies (1:1000). After washing with TBST, horseradish peroxidase-labelled secondary antibodies (1:2000) were added, and the blots were incubated for 2 h. Protein bands were activated by chemiluminescence and visualized on an X-ray film.

### 2.7. Transfection

Transfection was conducted as previously described with slight modifications [30]. Briefly, hepatocytes were seeded onto 6-well plates until 50% confluence and transfected using Lipofectamine 3000 (Invitrogen, Carlsbad, CA, USA). For overexpression of miR-195, a mimic or negative control was used at a concentration of 50 nM. HepG2 cells were treated with the miR-195 mimic in Opti-MEM medium for 24 h. The transfection medium was replaced with sorghum ethanol extract (5 and 10 μg/mL) or taxifolin (25 and 50 μM) in serum-free medium for 24 h. For inhibition of the miR-195, 50 nM of an inhibitor or negative control was used. hsa-miR-195-5p mimic (ThermoFisher Scientific, Inc., Worcester, MA, USA, Cat #4464066, Assay ID MC10827), hsa-miR-195-5p inhibitor (ThermoFisher Scientific, Inc., Worcester, MA, USA, Cat #4464084, Assay ID MH10827), miR mimic negative control (ThermoFisher Scientific, Inc., Worcester, MA, USA, Cat #4464058), and miR inhibitor negative control (ThermoFisher Scientific, Inc., Worcester, MA, USA, Cat #4464076) were used for transfection.

### 2.8. Quantitative Real-Time PCR

The total RNA was isolated using Trizol reagent (Invitrogen, Carlsbad, CA, USA) according to manufacturer’s instructions. To confirm miR-195 expression, the synthesized cDNA using TaqMan^TM^ microRNA reverse transcription kit (ThermoFisher Scientific, Inc., Worcester, MA, USA,) was analyzed using quantitative real-time PCR (Applied Biosystems, Carlsbad, CA, USA). The relative expression of hsa-miR-195-5p (ThermoFisher Scientific, Inc., Worcester, MA, USA, Cat #4427975, Assay ID 000494) was normalized to U6 snRNA (ThermoFisher Scientific, Inc., Worcester, MA, USA, Cat #4427975, Assay ID 001973).

### 2.9. Statistical Analysis

Data are presented as mean ± standard error from at least three independent experiments. Student’s t-test using SAS (version 8.1; SAS Institute, Cary, NC, USA) and Tukey’s post-hoc test using GraphPad Prism software 5.0 (GraphPad Software Inc., La Jolla, CA, USA) were used.

## 3. Results and Discussion

### 3.1. Identification of Taxifolin in Sorghum Ethanol Extract

**Figure 1:**
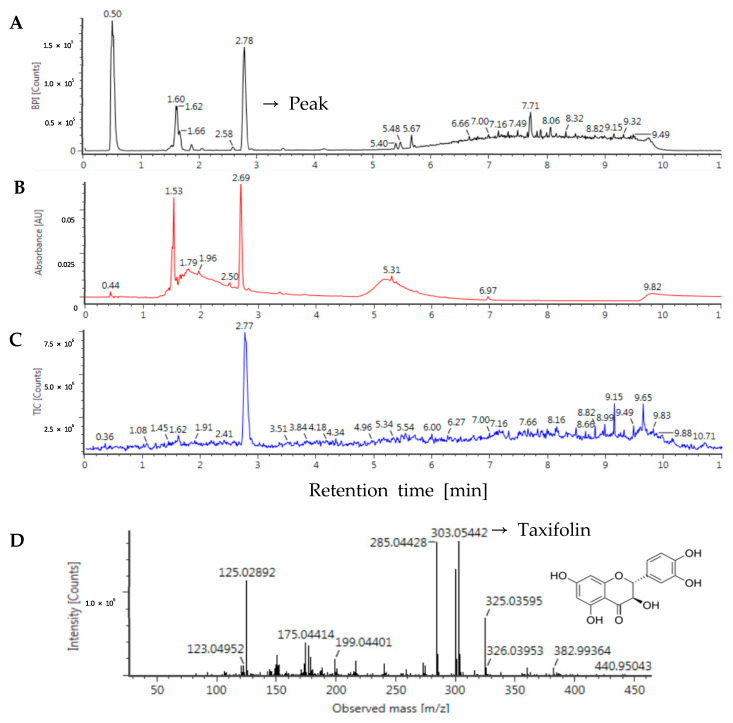
Chromatogram of sorghum ethanol extract obtained by ultra-performance liquid chromatography-electrospray ionization quadrupole time-of-flight mass spectrometry (UPLC-ESI-Q-TOF-MS). The total chromatogram of sorghum ethanol extract for (**A**) negative ion, (**B**) photodiode array (PDA) chromatogram, (**C**) the taxifolin standard, and (**D**) the fragmentation pattern.

The antidiabetic activity of plant materials correlates with the content of bioactive compounds such as flavonoids and polyphenols [31]. UPLC-ESI-Q-TOF/MS was used to determine the composition of sorghum ethanol extract according to its retention time, standard, and pseudo-molecular ion formation ([M − H]^−^). According to the UPLC and PDA chromatograms (at λ = 280 nm) of the sorghum ethanol extract shown in Figure 1, the peak was detected at a retention time of 2.78 min with formula of C_15_H_12_O_7_, neutral mass (Da) of 304.0583, observed ion (*m*/*z*) of 303.0546, and adduct of −H. Based on the MS/MS spectra and comparisons of the proposed fragment pattern in previous studies [32,33], the peak was identified as taxifolin. Taxifolin content (195 mg/100 g sorghum) was calculated by plotting a calibration curve using a standard. Taxifolin is a unique dihydroflavonol, which is the main flavonoid detected in sorghum. A recent study reported that the taxifolin content in sorghum grains ranges from 1.37 to 44.62 mg/100 g dry weight [1]. In recent years, because of its medicinal value, taxifolin has been widely used in the treatment of atherosclerosis, dyslipidemia, cardiovascular diseases, and other chronic diseases. Taxifolin treatment did not induce any apparent systemic abnormalities in TgSwDI mice [34]. A previous study showed that taxifolin at 25 mg/kg (equivalent to 240 mg/60kg human) can improve homeostasis of glucose in male spontaneously hypertensive (SHR) rats [11]. The absolute bioavailability of taxifolin was reported as 0.17% in rats. The bioavailability of taxifolin was 36% in rabbits upon detection of total conjugated and free taxifolin in plasma following enzymatic hydrolysis [35]. In the metabolite study, 191 metabolites from taxifolin were identified and 17 metabolites among them had similar bioactivities to taxifolin. This indicated that the effective form of taxifolin is the metabolites arising from it in vivo as well as the parent form. Moreover, taxifolin and its metabolites including quercetin, isorhamnetin disulfate, eriodictyol, luteolin-7-O-glucuronide, 3/4-hydroxyphenylpropionic acid, dihydroxyphenylacetic acid, and dihydrocaffeic acid have been reported to exert similar anti-diabetic activity [35]. Therefore, taxifolin and sorghum ethanol extract could be promising candidates for the treatment or prevention of diabetes.

### 3.2. Effects of Taxifolin and Sorghum Ethanol Extract on Cell Viability and Glucose Uptake

**Figure 2:**
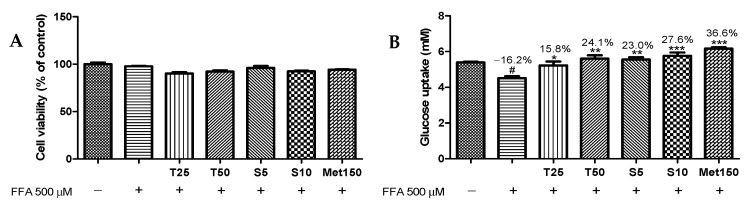
Effects of taxifolin and sorghum ethanol extract on (**A**) cell viability and (**B**) glucose uptake in HepG2 cells. Data are presented as the mean ± standard error (n = 3). ^#^ *p* < 0.05 versus the control cells; * *p* < 0.05, ** *p* < 0.01, and *** *p* < 0.001 versus the FFA-treated group. FFA, free fatty acid; T, taxifolin (μM); S, sorghum ethanol extract (μg/mL); Met, metformin (μM).

Inducing glucose absorption in the tissues and simultaneously supplying antioxidants and α-glucosidase inhibitors through nutrients could be a feasible and potential strategy to manage T2D [36]. Several studies showed that cereal grains and their phytochemicals possess antidiabetic activity. Polyphenols improve glucose consumption and upregulate insulin-dependent signals in many cell types [31]. Chung et al. (2011) reported that sorghum extract decreases serum fasting glucose and cholesterol levels [37]. Treatment with taxifolin (25 and 50 μM) or sorghum ethanol extract (5 and 10 μg/mL) along with FFA (500 μM) did not result in significant changes in cell viability (Figure 2A). As shown in Figure 2B, FFA treatment significantly reduced glucose uptake by 16.2% compared with that in the control. Exposure to taxifolin (50 μM) and sorghum ethanol extract (10 μg/mL) enhanced glucose uptake by 24.1% and 27.6%, respectively, compared with that with the FFA treatment. Our results indicated that taxifolin and sorghum ethanol extract exerts an important role in the consumption of glucose in insulin resistance conditions.

### 3.3. Effects of Taxifolin and Sorghum Ethanol Extract on the IRS1/PI3K/AKT and AMPK Signalling Pathways

**Figure 3:**
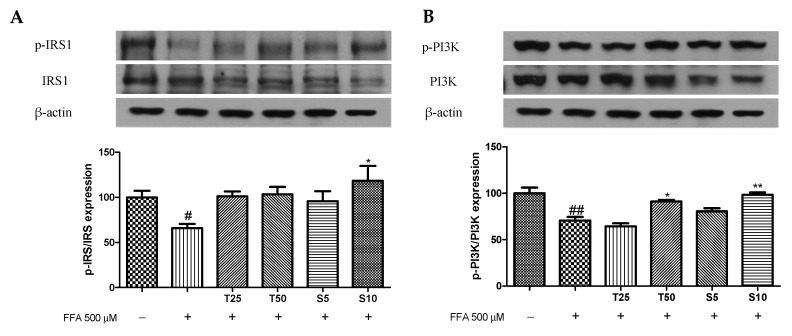

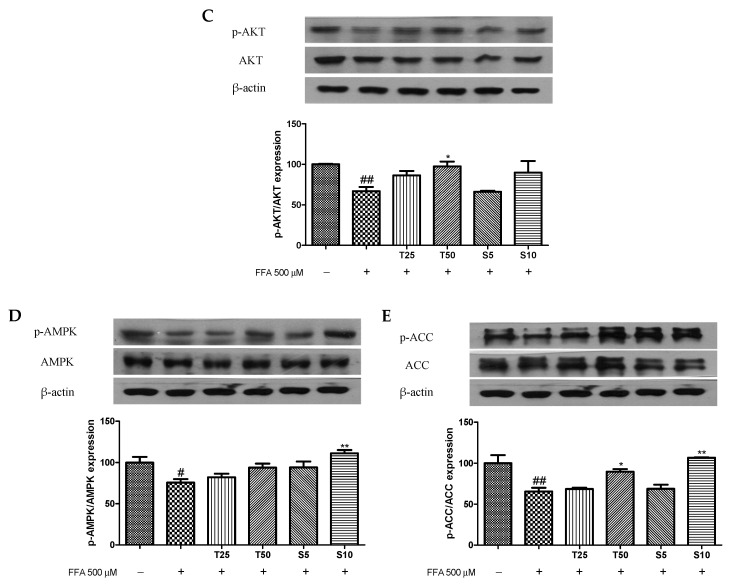
Effects of taxifolin and sorghum ethanol extract on phosphorylation of (**A**) IRS1, (**B**) PI3K, (**C**) AKT, (**D**) AMPK, and (**E**) ACC in HepG2 cells. Data are presented as the mean ± standard error (n = 3). ^#^ *p* < 0.05, ^##^ *p* < 0.01 versus the control cells; * *p* < 0.05, ** *p* < 0.01 versus the FFA-treated group. FFA, free fatty acid; T, taxifolin (μM); S, sorghum ethanol extract (μg/mL).

The PI3K/AKT signaling pathway is a classic insulin signaling pathway that can modulate glucose transport by activating insulin [38]. AMPK is an evolutionarily conserved serine/threonine kinase whose activation induces insulin-sensitizing effects, making it an ideal therapeutic target for T2D [39]. Administration of sorghum extract significantly decreased the expression of p-p38 and PEPCK while increasing p-AMPK expression [40]. Gao et al. (2020) showed that taxifolin modulates insulin signaling in the kidneys via the PI3K/AKT pathway [11]. To investigate the effects of taxifolin and sorghum ethanol extract on the insulin signaling pathway, we confirmed IRS1, PI3K, AKT, AMPK, and ACC phosphorylation levels by western blotting. Compared with the control, FFA treatment in hepatocytes reduced the phosphorylation of IRS1^tyr^ (Figure 3A). However, sorghum ethanol extract significantly increased IRS1^tyr^ phosphorylation at concentration of 10 μg/mL. Similarly, PI3K and AKT phosphorylation levels were decreased following FFA treatment in hepatocytes, whereas treatment with taxifolin and sorghum ethanol extract rescued the reduced phosphorylation in a dose-dependent manner (Figure 3B,C). As shown in Figure 3D,E, the phosphorylation of AMPK was significantly enhanced by sorghum ethanol extract (10 μg/mL) administration and the phosphorylation of ACC was increased by taxifolin (50 μM) and sorghum ethanol extract (10 μg/mL). Consistent with the findings of previous reports, regulation of the IRS1/PI3K/AKT and AMPK pathways is a possible mechanism by which sorghum ethanol extract alleviates insulin resistance.

### 3.4. Effects of Taxifolin and Sorghum Ethanol Extract on IRS1, PI3K, AKT, AMPK, and ACC Phosphorylation in miR-195 Mimic- and Inhibitor-Transfected Cells

**Figure 4:**
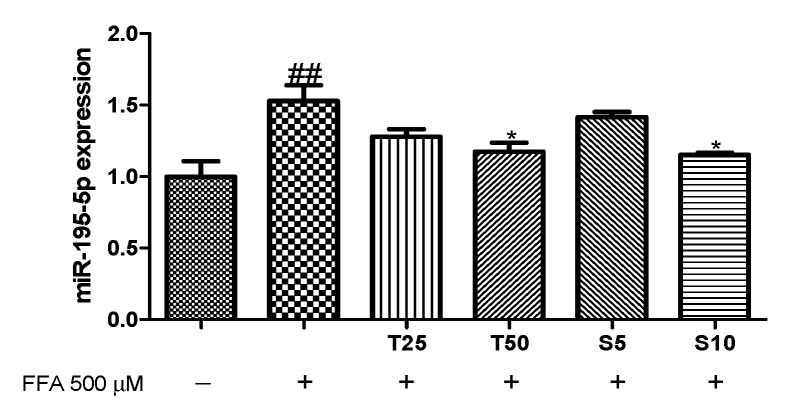
Effects of taxifolin and sorghum ethanol extract on the miR-195 expression in HepG2 cells. Data are presented as the mean ± standard error (n = 3). ^##^ *p* < 0.01 versus the control cells. * *p* < 0.05 versus the FFA-treated group. FFA, free fatty acid; T, taxifolin (μM); S, sorghum ethanol extract (μg/mL).

miR-195 is known to directly target the insulin receptor, which is IRS1 upstream [22]. Searching the TargetScan (http://www.targetscan.org accessed on 1 September 2020) database reveals putative miR-195-binding sites on the 3′-UTR of IRS1, PI3K, AMPK, and ACC mRNA. To evaluate the involvement of miR-195 in the effects of taxifolin and sorghum ethanol extract on the modulation of the IRS1/PI3K/AKT signaling pathway in the FFA-treated HepG2 cells, we confirmed the changes in miR-195 expression levels and investigated p-IRS1, p-PI3K, p-AKT, p-AMPK, and p-ACC expression levels. As shown in Figure 4, FFA treatment increased the miR-195 level. However, treatment with taxifolin or sorghum ethanol extract decreased miR-195 levels in a dose-dependent manner. We overexpressed or inhibited miR-195 using a mimic (50 nM) or an inhibitor (50 nM), respectively. The miR-195 level was significantly enhanced in overexpressed cells and decreased in inhibited cells compared with that in negative control-treated cells (Figure S1).

**Figure 5:**
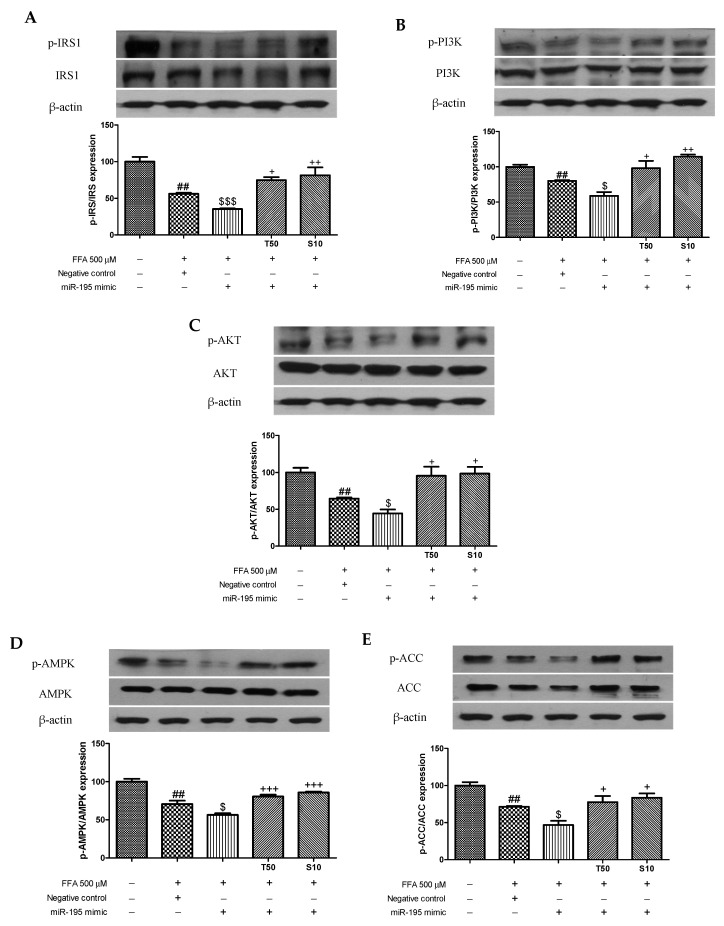
Transfection with the miR-195 mimic partially prevents taxifolin- and sorghum ethanol extract-mediated regulation of the IRS1/PI3K/AKT and AMPK signaling pathways in HepG2 cells following the FFA treatment. Relative (**A**) p-IRS1, (**B**) p-PI3K, (**C**) p-AKT, (**D**) p-AMPK, and (**E**) p-ACC protein levels in HepG2 cells transfected with the miR-195 mimic (50 nM) or its negative control (50 nM). Data are presented as the mean ± standard error (n = 3). ^##^ *p* < 0.01 versus the control cells; ^$^ *p* < 0.05, and ^$$$^ *p* < 0.001 versus the FFA+negative control-treated cells; ^+^ *p* < 0.05, ^++^ *p* < 0.01, and ^+++^ *p* < 0.001 versus the miR-195 mimic-treated cells. FFA, free fatty acid; T, taxifolin (μM); S, sorghum ethanol extract (μg/mL).

**Figure 6:**
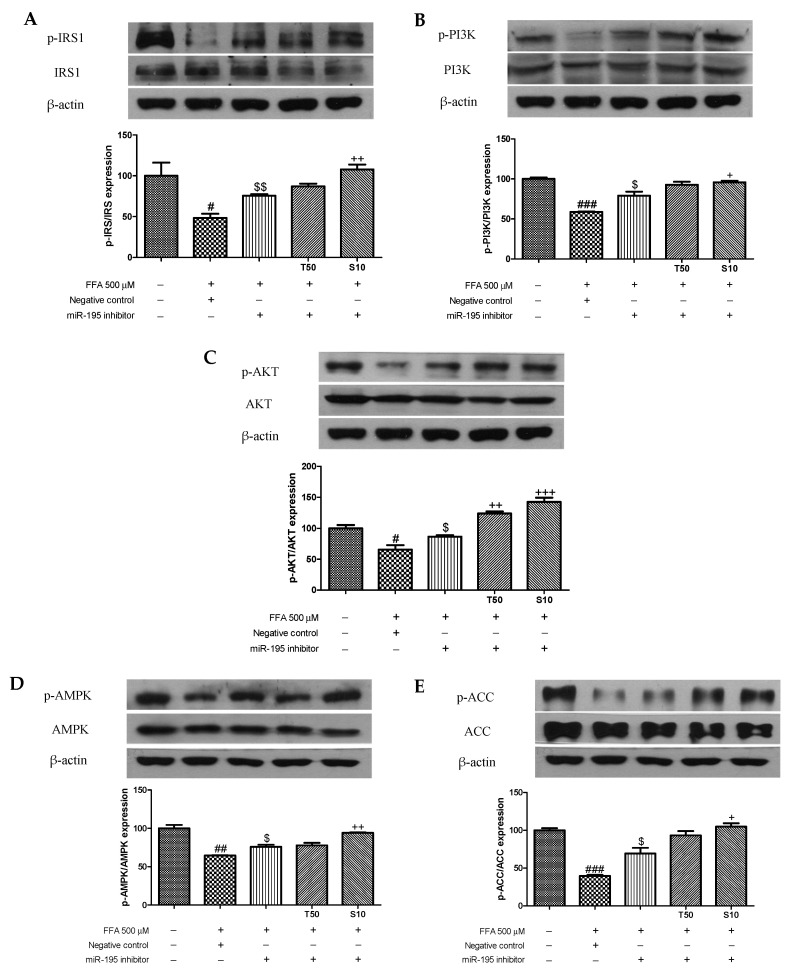
Transfection with the miR-195 inhibitor partially prevents taxifolin- and sorghum ethanol extract-mediated regulation of the IRS1/PI3K/AKT and AMPK signaling pathways in HepG2 cells following the FFA treatment. Relative (**A**) *p*-IRS1, (**B**) p-PI3K, (**C**) p-AKT, (**D**) p-AMPK, and (**E**) p-ACC protein levels in HepG2 cells transfected with the miR-195 inhibitor (50 nM) or its negative control (50 nM). Data are presented as the mean ± standard error (n = 3). ^#^ *p* < 0.05, ^##^ *p* < 0.01, and ^###^ *p* < 0.001 versus the control cells; ^$^ *p* < 0.05, and ^$$^ *p* < 0.005 versus the FFA+negative control-treated cells; ^+^ *p* < 0.05, ^++^ *p* < 0.01, and ^+++^ *p* < 0.001 versus the miR-195 inhibitor-treated cells. FFA, free fatty acid; T, taxifolin (μM); S, sorghum ethanol extract (μg/mL).

Overexpression or inhibition of miR-195 affected the phosphorylation of IRS1, PI3K, AKT, AMPK, and ACC. Transfection with the miR-195 mimic partly aggravated the FFA-induced downregulation of p-IRS1 (Figure 5A). However, taxifolin and sorghum ethanol extract enhanced the phosphorylation of IRS1. Similarly, PI3K, AKT, AMPK, and ACC phosphorylation levels were inhibited following FFA with miR-195 mimic treatment, whereas treatment with sorghum ethanol extract significantly rescued the downregulated phosphorylation (Figure 5B–E). In miR-195 inhibitor-transfected HepG2 cells, FFA treatment partially increased the phosphorylation of IRS1, PI3K, AKT, AMPK, and ACC (Figure 6). Taxifolin and sorghum ethanol extract significantly increased p-IRS1, p-PI3K, p-AKT, p-AMPK, and p-ACC levels in FFA-treated cells transfected with the miR-195 inhibitor. These results demonstrated that taxifolin and sorghum ethanol extract might reduce miR-195 expression levels to partially alleviate FFA-induced dysregulation of insulin resistance. There is evidence that miRs are associated with the progression of insulin resistance. Yang et al. (2014) reported that miR-195 is involved in insulin signaling and glycogen metabolism [23]. A recent study found that miR-138-5p regulates T2D progression by inducing autophagy in hepatocytes [41]. Compared with the insulin resistance group, miR-93-5p overexpression markedly increased glucose uptake, cell proliferation, and glycogen synthesis [42]. Celastrol reversed palmitic acid-induced insulin resistance by restoring the miR-223 and GLUT4 pathways [43]. Shu et al. (2020) reported that resveratrol significantly increased p-PI3K and p-AKT expression levels in miR-363 mimic-transfected HepG2 cells. However, the miR-363 inhibitor partially blocked resveratrol-mediated upregulation of the PI3K-AKT signaling pathway [44]. Zhao et al. (2017) showed that miR-124 mimic decreased glucose uptake and miR-124 inhibitor enhanced glucose consumption [45]. Especially, miR-195 expression levels correlating with glycaemic status are upregulated in a spontaneous rat model of T2D [23]. In patients with metabolic syndrome and T2D, miR-195 was identified as a potential contributor [24]. miR-195 regulates SIRT1-mediated changes in diabetic retinopathy [46]. Our findings are similar to those of several studies in which miR-195 was elevated in the liver of diabetic animals and patients. However, downregulation of miR-195 by taxifolin and sorghum ethanol extract may protect against insulin resistance in HepG2 cells. There is an enormous amount of evidence concerning the diverse role of miRs in various pathological conditions and miRs have been associated with many cellular processes for various signaling pathways [47,48,49,50,51,52,53,54].

## 4. Conclusions

The present study showed that taxifolin and sorghum ethanol extract attenuated FFA-induced hepatic insulin resistance by suppressing miR-195 expression. Sorghum is a rich source of phytochemicals such as phenolic acids and flavonoids, which may contribute to its antidiabetic activity. Moreover, taxifolin is a bioactive flavonoid of dietary origin that performs several functions. The antidiabetic activity of taxifolin and sorghum ethanol extract was demonstrated through the regulation of miR-195 expression and the IRS1/PI3K/AKT, and AMPK signaling pathways. Hence, our results suggest that taxifolin and sorghum ethanol extract may be useful alternatives to miR-based therapeutics.

## Data Availability

Data is contained within the article and supplementary material.

## Supplementary Materials

The following are available online at https://www.mdpi.com/article/10.3390/antiox10091331/s1, Figure S1: Overexpression and silencing of microRNA-195: miR-195 expression was significantly increased by transfection with (A) the miR-195 mimic and decreased by transfection with (B) the miR-195 inhibitor.
