# Supplementary material for: Taxifolin and Sorghum Ethanol Extract Protect against Hepatic Insulin Resistance via the miR-195/IRS1/PI3K/AKT and AMPK Signalling Pathways
Source: Antioxidants (Basel). 2021 Aug 24;10(9):1331. doi: 10.3390/antiox10091331 (PMC8465682; doi:10.3390/antiox10091331)
Supplement: Supplementary file 1 [file antioxidants-10-01331-s001.zip › antioxidants-1331494-supplementary.pdf]

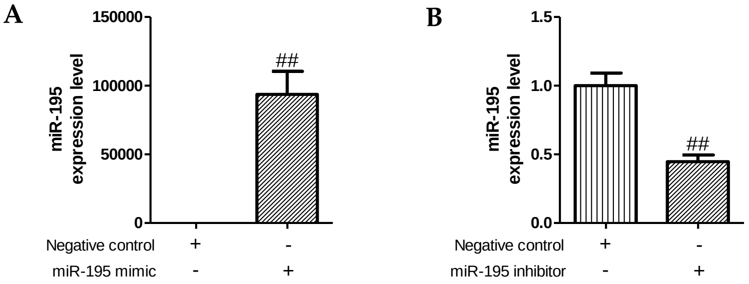

Figure S1. Overexpression and silencing of microRNA-195: miR-195 expression was significantly increased by transfection with (A) the miR-195 mimic and decreased by transfection with (B) the miR-195 inhibitor. ##*P*<0.01 versus the negative control cells.
